# 

**Published:** 1889-07

**Authors:** 


JULY, 1889.

### THE AMERICAN JOURNAL OF DENTAL SCIENCE.

LIBRARY INDEXED S.G.O.

PUBLISHED MONTHLY.

$2.50 PER ANNUM.

EDITED BY

F. J. S. GORGAS, M. D., D. D. S.

Vol. 23. THIRD SERIES. No. 3.

BALTIMORE:
SNOWDEN & COWMAN, 9 W. FAYETTE STREET.

LONDON:
TRUBNER & CO., 60 PATERNOSTER ROW.

PAYABLE IN ADVANCE.

